# Intraspecific and interspecific trait variability in tadpole meta‐communities from the Brazilian Atlantic rainforest

**DOI:** 10.1002/ece3.5031

**Published:** 2019-03-13

**Authors:** Mainara Xavier Jordani, Nicolas Mouquet, Lilian Casatti, Marcelo Menin, Denise de Cerqueira Rossa‐Feres, Cécile Hélène Albert

**Affiliations:** ^1^ Programa de Pós‐graduação em Biologia Animal Universidade Estadual Paulista (UNESP) São José do Rio Preto Brazil; ^2^ Univ Montpellier, CNRS, Ifremer, IRD MARBEC Montpellier France; ^3^ Departamento de Zoologia e Botânica Universidade Estadual Paulista (UNESP) São José do Rio Preto Brazil; ^4^ Departamento de Biologia Universidade Federal do Amazonas (UFAM) Manaus Brazil; ^5^ Laboratório de Ecologia Teórica, Departamento de Zoologia e Botânica Universidade Estadual Paulista (UNESP) São José do Rio Preto Brazil; ^6^ Aix Marseille Univ Univ Avignon, CNRS, IRD, IMBE Marseille France

**Keywords:** community assembly, ecological niche, fitness differences, phenotypic variability, stabilizing niche differences

## Abstract

1. A better understanding of species coexistence and community dynamics may benefit from more insights on trait variability at the individual and species levels.
2. Tadpole assemblages offer an excellent system to understand the relative influence of intraspecific and interspecific variability on community assembly, due to their high phenotypic plasticity, and the strong influence that environmental variables have on their spatial distribution and individual performance.
3. Here, we quantified the intraspecific and interspecific components of tadpoles’ trait variability in order to investigate their relative role in shaping tadpole communities.
4. We selected eight functional traits related to microhabitat use, foraging strategies, and swimming ability. We measured these traits on 678 individuals from 22 species captured in 43 ponds in the Atlantic Forest. We used single‐ and multitrait analyses to decompose trait variability. To explore the action of external and internal filtering on community assembly, we used a variance decomposition approach that compares phenotypic variability at the individual, population, community and regional levels.
5. On average, 33% of trait variability was due to within‐species variation. This decomposition varied widely among traits. We found only a reduced effect of external filtering (low variation in the height of the ventral fin within ponds in comparison to the total variation), whereas the internal filtering was stronger than expected. Traits related to the use of different microhabitats through the water column were generally less variable than traits related to swimming ability to escape of predators, with tail traits being highly variable within species.
6. Our study highlights the importance of incorporating both intraspecific and interspecific, trait differences and of focusing on a diversity of traits related to both stabilizing niche and fitness differences in order to better understand how trait variation relates to species coexistence.

## 1 INTRODUCTION

For decades, ecologists have been interested in understanding how species trait differences drive the assembly of communities. Though intraspecific trait variation (hereafter, ITV) is at the origin of the theory of evolution, it has for a while been overlooked in trait‐based ecology, that was searching for big patterns among species (Hart, Schreiber, & Levine, 2016; Layman, Newsome, & Gancos, 2015; McGill, Enquist, Weiher, & Westboy, 2006). ITV combines genetic diversity and phenotypic plasticity, and is assumed to play a major role in community assembly processes (Violle et al., 2012). For instance, ITV can mediate species coexistence (Turcotte & Levine, 2016), determine the ability of natural systems to cope with environmental changes (González‐Suárez & Revilla, 2013; Jung et al., 2014; Laurila, Karttunen, & Merilä, 2002), and control competitive or trophic interactions (Hughes, Hanley, Orozco, & Zerebecki, 2015; Le Bagousse‐Pinguet et al., 2015; Zhao, Villéger, Lek, & Cucherousset, 2014).

These recent findings have led to general ecological theories being revisited in the light of ITV (Hart et al., 2016; Turcotte & Levine, 2016). After environmental filtering has excluded poorly adapted species, species coexistence is thought to be determined by a balance between two major opposing mechanisms: species fitness differences that drive competitive exclusion, and stabilizing niche differences that favor coexistence (Chesson, 2000). Traits related to fitness differences are those that favor one competitor over the other regardless of their relative abundance, while traits related to stabilizing niche differences are those that cause intraspecific interactions to be more limiting than interspecific interactions (Chesson, 2000; Kraft et al., 2015). By altering traits related to both mechanisms, ITV could enhance or impede species coexistence (Turcotte & Levine, 2016). However, it remains unclear what the general outcome might be. Turcotte and Levine (2016) found some evidence that ITV impact on stabilizing niche differences may either enhance coexistence by reducing interspecific competition (e.g., shift in resource acquisition traits) or impede coexistence by leading to trait convergence and increased niche overlap. ITV impact on average fitness differences may also enhance species coexistence by minimizing the competitive disadvantages of subordinate species in competitive asymmetries (Le Bagousse‐Pinguet, Bello, Vandewalle, Leps, & Sykes, 2014), but this still has little empirical support (Turcotte & Levine, 2016).

Some tools have now been developed to better encompass ITV in community ecology (Carmona et al., 2012; Lepš, Bello, Šmilauer, & Doležal, 2011; Violle et al., 2012). The new *T*‐statistics variance ratios framework is one of these tools developed to analyze trait patterns in order to infer hypotheses about community assembly processes while accounting for ITV (Taudière & Violle., 2016; Violle et al., 2012). By comparing the variance of trait values at different scales (population, community, regional pool), this framework allows detection of the signature of external (sorting of species from a regional pool due to broad‐scale gradients) and internal (microenvironmental heterogeneity and biotic interactions) filters on local trait value distributions, while accounting or not for ITV (Violle et al., 2012). Recent studies have suggested that disentangling multiple functional diversity components may provide a better understanding of the processes involved in the structure of plant communities (Le Bagousse‐Pinguet et al., 2014). To date, research on ITV and community assembly has mainly focused on plants (but see Zhao et al., 2014; Griffiths, Louzada, Bardgett, & Barlow, 2016). For example, the *T*‐statistics variance ratios framework has already been applied to detect the effects of external and internal filters on the assembly of plant communities across soil (Le Bagousse‐Pinguet et al., 2014) and elevation gradients (Neyret et al., 2016).

Anuran tadpole meta‐communities have been broadly used as a model system to study competition (Richter‐Boix, Llorente, & Montori, 2007), and offer an excellent system to explore the role of ITV in trait‐based community assembly processes. Firstly, tadpoles have been found to exhibit extensive trait phenotypic variability (e.g., morphology, physiology and behavior), in response to both physical environment (Eterovick & Barata, 2006; Eterovick, Lazarotti, Franco, & Dias, 2010), and density of predators and competitors (Michel, 2012; Relyea, 2005). Secondly, tadpoles represent the major part of the biomass in freshwater habitats (Altig, Whiles, & Taylor, 2007), being important primary and secondary consumers, and therefore are known to play a major role in ecosystem functioning (Strauβ, Reeve, Randrianiaina, Vences, & Glos, 2010). However, we still know little regarding the assembly of natural communities of amphibians at larval stage (Grözinger, Thein, Feldhaar, & Rödel, 2014; Strauβ et al., 2010; Zhao, Li, Wang, Xie, & Jiang, 2017).

Here, we use a trait‐based approach on tadpole communities to quantify the intraspecific and interspecific components of tadpole trait variation, and infer hypotheses regarding the role of ITV in the assembly of these communities on the basis of the *T*‐statistics variance ratios framework (Violle et al., 2012). By analyzing a set of eight traits measured on individual tadpoles in 43 ponds in the Atlantic Forest, we address two main questions:

1. How is trait variation structured within and among species?
2. What is the importance of external and internal filters in shaping the assembly of tadpole communities, and what is the contribution of ITV to these processes? Several studies have indicated that biotic interactions have less influence in shaping tadpole communities than environmental or stochastic factors (Kopp & Eterovick, 2006; Strauβ et al., 2010). Tadpoles can also use a great diversity of microhabitats and exhibit high levels of functional redundancy (Eterovick, 2003; Grözinger et al., 2014; Richter‐Boix et al., 2007; Strauβ et al., 2010). We thus expect external filters to play a major role in the communities’ structure and internal filters to be less strong, with high trait overlap among populations within a community.

From our results, we infer hypotheses on how the different levels of ITV found for the different traits we measured may promote coexistence within tadpole communities. We assume traits related to stabilizing niche differences—that is, traits related to the use of microhabitats, such as those determining the individual's position in the water column—to be less variable within species than those related to fitness differences—that is, the traits related to foraging strategies and swimming capacity (Turcotte & Levine, 2016).

## 2 MATERIALS AND METHODS

### 2.1 Study area

The Atlantic Rain Forest is a biodiversity hotspot (Myers, Mittermeier, Mittermeier, Fonseca, & Kent, 2000) and a Biosphere Reserve (UNESCO). Its lowlands are distributed from 5 to 50 m above sea level, between 16° to 24° latitude south (IBGE, 2012), across the coastal plains of Brazil. The vegetation is characterized by macro‐ and meso‐phanerophytes, lianas and epiphytes (IBGE, 2012). We sampled communities in four different localities (Ubatuba, Bertioga, Itanhaém, and Iguape municipalities) in São Paulo state, southeastern Brazil (Supporting information Figure S1). Bertioga, Iguape and Itanhaém have a tropical climate, with no dry season (*Af* in Köppen‐Geiger classification, Alvares, Stape, Sentelhas, Gonçalves, & Sparovek, 2013). The climate in Ubatuba is humid subtropical, with influence of the oceanic climate and hot summers (*Cfa *in Köppen‐Geiger classification, Alvares et al., 2013). Both climates are characterized by high temperatures (mean annual temperature: *Af* = 21.5°C; *Cfa *= 20.8; Alvares et al., 2013) and high rainfall evenly distributed throughout the year (cumulative annual rainfall: *Af* = 2,309 mm; *Cfa* = 2,243 mm; Alvares et al., 2013).

### 2.2 Database description

A standardized protocol was used to sample tadpole communities and characterize 43 ponds in the Atlantic Forest in 2011–2013. Tadpoles were sampled using a hand dip‐net (32 cm diameter) with a 3 mm^2^ mesh. Dip‐net surveys were carried out throughout the total area of aquatic habitats, to ensure a good representation of all the microhabitats (Rossa‐Feres & Jim, 1996; Skelly & Richardson, 2010). The ponds sampled represent the wide variety of environmental conditions in the study area, presenting contrasting size, water depth, amount of aquatic vegetation, substrate types on the bottom, and percentage of canopy cover (Supporting information Table S1). All collected tadpoles were conserved in a solution of alcohol 70% and formaldehyde 15% (1:1) and deposited in a scientific collection (DZSJRP Amphibia‐Tadpoles collection. Department of Zoology and Botany, Universidade Estadual Paulista, São José do Rio Preto, São Paulo, Brazil).

We measured traits on 1 to 20 individuals for each species occurring in each pond. For logistical reasons, and due to the aggregative behavior of some species that results in very disparate abundances (very high or very low, Hoff, Blaustein, McDiarmid, & Altig, 1999), we picked 20 as a manageable maximum number of individuals per species per pond. Individuals were all at the developmental stages from 27 to 37 (Gosner, 1960). This phase is considered to be a developmental “climax” period, when changes in body parts of the tadpoles are isometric, and when they are best suited to morphological intraspecific and interspecific comparisons, with a lesser impact of ontogeny (Gosner, 1960; Grosjean, 2005; Wassersug, 1973).

We thus measured traits on 678 tadpoles from 22 anuran species (Supporting information Table S2). This full dataset was used to investigate the structuration of trait variation within and among species (question 1). To assess the generality of our results with regard to question 1, we also used a supplementary dataset from the Amazon Forest, that is, a different biome (see Supplementary Material). This dataset contains 60 tadpoles from 13 species (Supporting information Table S3), collected (similar protocols as for the Atlantic forest, except that the development stages are from 33 to 37) in 31 ponds in three different localities (Iranduba, Manaus, and Presidente Figueredo cities) in Amazonas state (Northern Brazil).

To investigate the importance of external and internal filters in the assembly of tadpole communities (question 2), we included only the tadpole communities with more than one species and more than one individual per species, which are the minimum requirements for calculation of the *T*‐statistics. Thus, for this second question, we reduced the dataset to a subset of 240 tadpoles from 18 species collected in 11 ponds.

### 2.3 Functional traits

**Figure 1:**
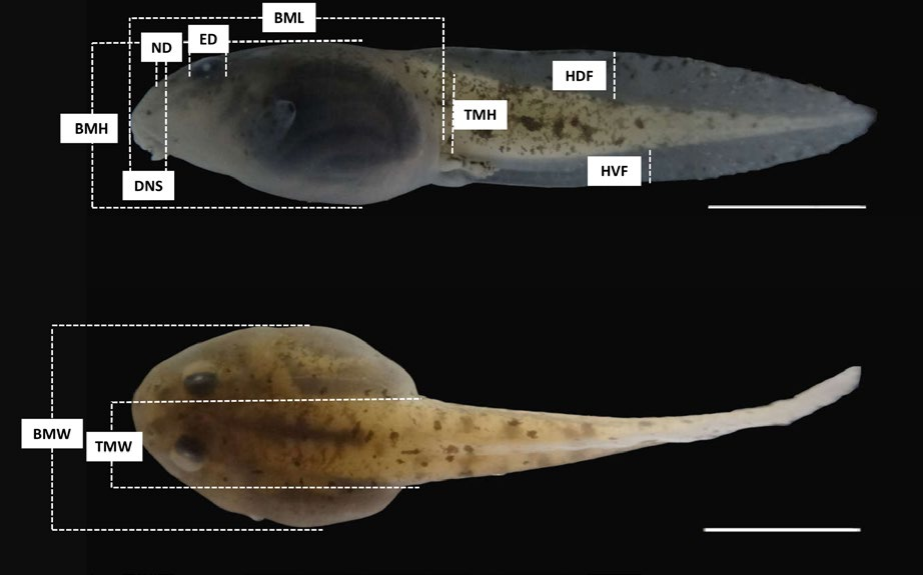
Visual representation of the 10 external morphological features of tadpoles used to determine the 8 tadpole traits (BML: body maximum length; BMH: body maximum height; BMW: body maximum width; DNS: distance from nares to snout; ED: eye diameter; HDF: maximum height of dorsal fin; HVF: maximum height of ventral fin; ND: nares diameter; TMW: tail muscle width; TMH: tail muscle height). Species: *Crossodactylus caramaschii*. Scale: 10mm

Eight functional traits were obtained from the 10 morphological features we measured on each tadpole (Table 1, Figure 1), following Altig and McDiarmid (1999) and Provete et al. (2013). All morphological features were measured in millimeters under a stereoscopic microscope with ocular micrometer (Leica MZ75), and were conducted by the same person to ensure consistency. For all traits, we used relative measures to control as much as possible for the ontogenetic variability (Supporting information Figure S2). The traits considered here are known to be related to essential elements of the ecology of tadpoles, namely resource use, position in the water column (Alford, 1999; Altig & Johnston, 1989; Van Buskirk, 2009; Hoff et al., 1999), body hydrodynamics, swimming ability (Altig & McDiarmid, 1999), feeding behavior (Altig & Johnston, 1989; Harris, 1999), and chemical perception (Altig & McDiarmid, 1999).

**Table 1:** Functional traits measured on individual tadpoles from Atlantic Forest

Abbreviations	Trait index description	Biological interpretation	Ecological function
BCI	Body compression index = body maximum height (BMH)/body maximum width (BMW)	Higher values indicate globular body, and lower values, depressed body	Variations in tadpoles body linked with variations in the height of fins determine the water column position used by tadpoles (Altig & Johnston, 1989; Altig & McDiarmid, 1999; Van Buskirk, 2009)
RDE	Relative diameter of the eyes = eye diameter (ED)/body maximum length (BML)	Higher values indicate bigger eyes, and lower, smaller eyes	Relates to tadpoles’ ability to perceive predators (mainly fish) in water bodies with different turbidity levels (Altig & Johnston, 1989; Altig & McDiarmid, 1999)
HDF	Height of the dorsal fin = height maximum of dorsal fin (HDF)/body maximum height (BMH)	Higher values indicate higher fin, and lower values lower fin	Relates to tadpoles’ ability to move through the water column and keep nektonic tadpoles in equilibrium (Altig & Johnston, 1989; Altig & McDiarmid, 1999; Hoff & Wassersug, 2000)
HVF	Height of the ventral fin = height maximum of ventral fin (HVF)/body maximum height (BMH)	Higher values indicate higher fin, and lower values lower fin	Relates to tadpoles’ ability to move through the water column and keep nektonic tadpoles in equilibrium (Altig & Johnston, 1989; Altig & McDiarmid, 1999)
RWT	Relative width of the tail = tail muscle width (TMW)/body maximum width (BMW)	Higher values indicate broad tail muscle, and lower values, narrow tail muscle	Relates to tadpoles’ use of different microhabitats (position in the water column), swimming mechanisms and styles. It is also related to ability (burst speed) to escape from active predators (Altig & McDiarmid, 1999; Van Buskirk, 2009; Van Buskirk & Relyea, 1998)
TCI	Tail compression index = tail muscle height (TMH)/tail muscle width (TMW)	Higher values indicate compressed and thinner tail, and lower values, thicker tail	Relates to tadpoles’ use of different microhabitats (position in the water column), swimming mechanisms and styles (Altig & McDiarmid, 1999)
DNS	Distance from nares to snout (DNS)	Higher values indicate nares closer to eyes and lower values anterior nares (closer to snout)	Position of nares relates to chemical perception. Anterior nares facilitate the search for food resources and detection of chemicals cues of predators (Altig & McDiarmid, 1999)
RDN	Relative diameter of the nares = nares diameter (ND)/body maximum length (BML)	Higher values indicate bigger nares, and lower, smaller nares	Variations in nares diameter relates to chemical perception of smells due to, in bigger nares, circulates larger volume of water (Altig & McDiarmid, 1999)

### 2.4 Data analysis

#### 2.4.1 Decomposition of trait variation within and among species

In order to decompose the variation of traits within and among species, we followed Albert et al. (2010). As different numbers of individuals and populations have been sampled for each species, we used a resampling procedure to balance the data sets (equal number of individuals) to perform both single and multitrait analyses. Initially, we determined the smallest number of individuals measured within species (*n* = 6). The five species with less than six individuals were removed from the analyses (Supporting information Table S2). Then, we subsampled the original dataset by drawing randomly 1,000 balanced data subsets containing the smallest number of individuals per species (Albert et al., 2010).

For the single‐trait approach, we used linear mixed models with restricted maximum likelihood estimation (Albert et al., 2010; Messier, McGill, & Lechowicz, 2010). For each trait we constructed the models trait ~ (1|species) that included no fixed effect and species as random intercept. We also tested the spatial structure of ITV with models including both species and populations nested within localities as random intercepts [i.e., trait ~ (1|species) + (1|localities/ponds)]. The model structure corresponds to the incompletely nested structure of the sampling design; species are not nested within ponds, nor ponds within species, because the same species could be found in different ponds (Auger & Shipley, 2013). Results were expressed as relative proportions of variance at each level.

To decompose the variance for the eight traits simultaneously (multitraits approach), we used between‐groups and within‐groups principal components analyses (PCA; Albert et al., 2010). Species were used as the grouping factor in these analyses. Between‐groups (respectively within‐) PCA uses the correlation matrix based on group means (respectively centered on group means), and finds linear combinations of variable maximizing variance among groups (respectively within groups) instead of the overall variance (Dodélec & Chessel, 1991). The inertia calculated in a between‐groups PCA represents the part of the total variance due to the differences between groups (Dodélec & Chessel, 1991). Both analyses lead to an identification of the traits that are responsible for trait variability among and within species.

#### 2.4.2 Importance of external and internal filters in shaping tadpole communities

In order to assess the influence of external and internal filtering in shaping tadpole communities, and the importance of ITV in these processes, we calculated *T*‐statistics (Violle et al., 2012). Trait variation (from individual trait measurements) can be decomposed into six components: (a) variance of trait values among individuals within populations (σIP^2^); (b) variance of trait values among individuals within communities (σIC^2^); (c) variance of trait values among individuals within the regional pool (σIR^2^); (d) variance of population mean trait values within communities (σPC^2^); (e) variance of population mean trait values within the regional pool (σPR^2^); and (f) variance of community mean trait values within the regional pool (σCR^2^). *T*‐statistics are ratios of these components that depict how some subsets of the trait variance are organized across spatial scales and biological levels.

*T*_IP.IC_ is the ratio between the variance of trait values among individuals within populations (σIP^2^), and the variance of trait values among individuals within communities (σIC^2^). It quantifies the overlap among species trait distributions within communities while accounting for individual trait variation. High values of *T*_IP.IC_ (close to 1) indicate high trait overlap among coexisting species, and mean that processes that lead to trait differentiation within species (e.g., asymmetric competition) are stronger than processes that lead to trait differentiation among species (e.g., specialization in microhabitat); competition and specialization operate at the individual and not at the species level.

*T*_IC.IR _is the ratio between the variance of trait values among individuals within communities (σIC^2^), and the variance of trait values among individuals within the regional pool (σIR^2^). It quantifies the overlap among community trait distributions within the region, while accounting for individual trait variation. High values of *T*_IC.IR_ (close to 1) indicate strong trait overlap among communities, and can be interpreted as low levels of external filtering (e.g., operated by climate, water depth, predation pressure) at the individual level.

*T*_PC.PR _is the same ratio as *T*_IC.IR_, but with population‐level means. It quantifies the overlap among community trait distributions within the region without accounting for individual trait variation. High values of *T*_PC.PR_ (close to 1) can be interpreted as low levels of external filtering at the species level.

To test the significance of *T*‐statistics, observed values (*I*_obs_) were compared with values obtained with randomized data (*I*_sim_). Standardized effect sizes (SES) were calculated:

$$\text{SES}=\left(I_{\text{obs}}-I_{\text{sim}}\right)/S_{\text{sim}},$$

where *I*_sim_ and *S*_sim_ are respectively the mean value and the standard deviation of the randomized values (*n* = 1,000 randomizations). SES measure the number of standard deviations that differentiate the observed index from the mean index of the simulated communities (Gotelli & McCabe, 2002). Negative (respectively positive) SES values reflect *T*‐statistic values that are lower (respectively higher) than random expectations, thus indicating trait distribution overlap that is lower (respectively higher) than random expectations.

In the *cati* package, randomizations are adapted to each metric (Taudière & Violle, 2016): (a) for *T*_IP.IC_, individual trait values are permuted within communities, keeping species composition unchanged, but breaking the link between species and trait values (null model = “local”), (b) for *T*_IC.IR_, trait values are permuted for all individuals in all communities in the regional pool, keeping the number of individuals in each community unchanged, but breaking the link between species and trait values at regional scale (null model = “regional.ind”), (c) for *T*_PC.PR_, population‐level trait values are permuted within the region, keeping the number of populations in each community unchanged (null model = “regional.pop.”).

Significance of the effect, that is, departure from random values, is assessed for each trait and each metric with a bilateral test. We chose a 5% significance level, meaning significant observed values are below the 0.025 quantile of random values or above their 0.975 quantile.

To test the potential effect of pond species richness on the observed internal filtering, linear regression models were performed for each trait between *T*_IP.IC _and species richness.

All statistics analysis we performed in R 3.3.1 (R Core Team, 2016), using different packages: lme4 (Bates, Maechler, Bolker, & Walker, 2015) for mixed models, ade4 (Dray & Dufour, 2007) for multivariate analyses, and cati for the *T*‐statistics (Taudière & Violle, 2016).

## 3 RESULTS

### 3.1 Decomposition of trait variation within and among species

On average, single‐ and multitraits analyses led to similar results regarding the relative contribution of intraspecific (33% and 30%) and interspecific (66% and 70%) components to the total trait variation (Table 2). We found relatively similar results with the dataset from the Amazon (see Supporting information Appendix S1), in which the relative contributions were: 19% for intraspecific and 80% for interspecific trait variability.

**Table 2:** Decomposition of tadpole trait variation in intraspecific and interspecific components and including the effect of different spatial scales (within species, between species, between populations, and between regions)

Functional traits	Intraspecific variability	Interspecific variability	Spatial decomposition			
			Within species	Between species	Between ponds nested in localities	Between localities
Body compression index	0.182 [0.182–0.185]	**0.816 [0.814**–**0.818]**	0.200	**0.790**	0.009	0
Relative diameter of eyes	0.163 [0.160–0.165]	**0.836 [0.834**–**0.839]**	0.153	**0.830**	0.016	0
Height of the dorsal fin	**0.624 [0.620**–**0.629]**	0.376 [0.371–0.380]	**0.443**	0.397	**0.155**	0.004
Height of the ventral fin	0.343 [0.340–0.345]	**0.657 [0.655**–**0.659]**	0.245	**0.679**	0.062	0.012
Relative width of the tail	0.255 [0.254–0.257]	**0.744 [0.743**–**0.746]**	0.194	**0.730**	0.066	0.009
Tail compression index	**0.693 [0.687**–**0.699]**	0.306 [0.300 ‐ 0.313]	**0.636**	0.275	0.087	0.002
Distance from nares to snout	0.153 [0.152–0.155]	**0.846 [0.845**–**0.848]**	0.137	**0.823**	0.038	0
Relative diameter of nares	0.260 [0.259–0.263]	**0.739 [0.737**–**0.734]**	0.247	**0.687**	0.063	0.002
Average of single‐trait analyses	0.334 [0.332–0.337]	**0.665 [0.662**–**0.667]**				
Multitraits analyses	0.295 [0.294–0.296]	**0.705 [0.704**–**0706]**				

Relative proportions of variance are given for each of eight tadpole traits separately (after a resampling procedure), as an average across traits (single‐trait analysis), and for all traits together (multitraits analysis). Square brackets represent the 95% confidence intervals from the resampling procedure. The largest (intraspecific or interspecific) component is in bold.

For single‐trait analysis, the contribution of ITV to the total variability differed considerably among traits (16%–69%). While traits related to body shape were mostly variable among species, the height of dorsal fin (HDF) and the tail compression index (TCI) were more variable within than between species, with ITV reaching up to 69% of the total variance (Table 2). We also detected high ITV (>85%) in the tail compression index (TCI) for tadpoles from the Amazon (Supporting information Appendix S1). Regarding the spatial structure, we did not detect any variation among localities (Bertioga, Iguape, Itanhaém, Ubatuba). We found only a small contribution (0%–15%) of differences among ponds within localities, mainly for the height of dorsal fin (HDF) and the tail compression index (TCI; Table 2).

**Figure 2:**
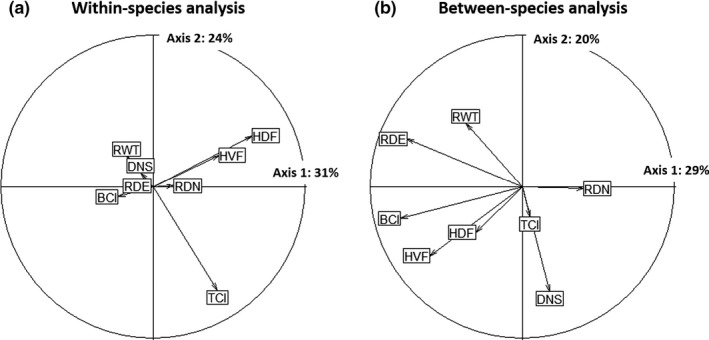
Correlation circles regarding the first two axes of the (a) within‐species and (b) between‐species PCA on functional traits for tadpole communities from Atlantic Forest. BCI: Body compression index; DNS: Distance from nares to snout; HDF: Relative height of the dorsal fin; HVF: Relative height of the ventral fin; RDE: Relative diameter of the eyes; RWT: Relative width of the tail; RDN: Relative diameter of the nares; TCI: Tail compression index

For multitrait analysis, the within‐species trait variability was mainly structured by a first axis (31% of variance) driven by the height of dorsal fin (HDF), and by a second axis (24% of variance) driven by the tail compression index (TCI; Figure 2a). Within species, individual tadpoles thus go from narrow dorsal fins and slightly compressed tails (top left quadrant, Figure 2a), to broad dorsal fins and highly compressed tails (bottom right quadrant), these representing different swimming abilities and position in the water column. Between‐species, trait variability was mainly structured by a first axis (29% of variance) driven by the body compression index (BCI), the relative diameter of eyes (RDE), and the height of the ventral fin (HVF), and by a second axis (20% of variance) driven by the distance from nares to snout (DNS) and the relative width of the tail (RWT; Figure 2b). Species thus go from highly to slightly globular bodies (first axis), and from nares close to the eye and narrow tail muscle, to nares distant from the eyes and large tail muscle (second axis; Figure 2b).

### 3.2 Importance of external and internal filters in shaping tadpole communities

**Figure 3:**
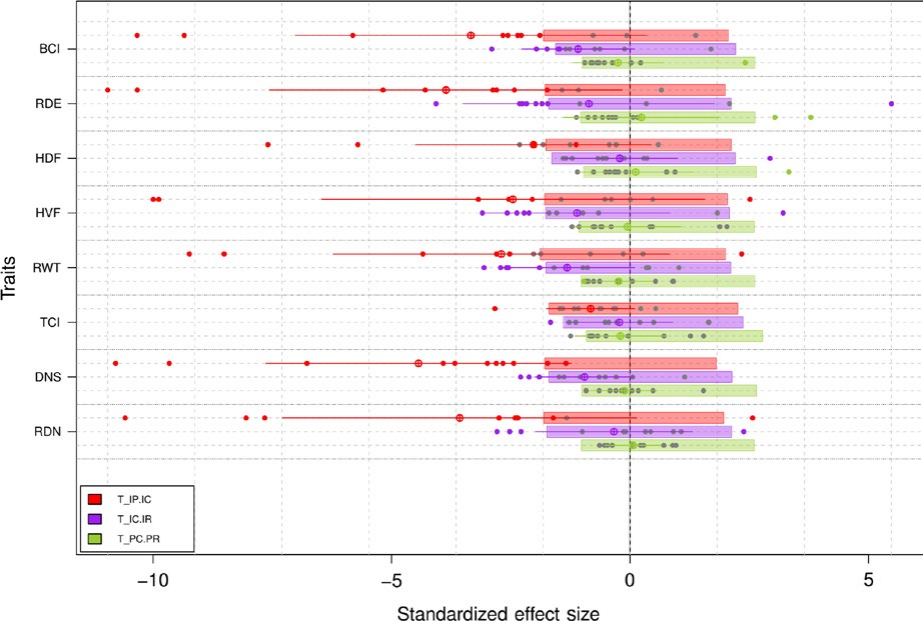
Standardized effect size (SES) of *T*‐statistics for eight tadpole functional traits (BCI: Body compression index; DNS: Distance from nares to snout; HDF: Relative height of the dorsal fin; HVF: Relative height of the ventral fin; RDE: Relative diameter of the eyes; RWT: Relative width of the tail; RDN: Relative diameter of the nares; TCI: Tail compression index). Three *T*‐statistics are given: (a) *T*_IP.IC_— the within‐population variance relative to the total variance in the community; (b) *T*_IC.IR_—community‐wide variance relative to the total variance in the regional pool, assessed at the individual level; (c) *T*_PC.PR_—community‐wide variance relative to the total variance in the regional pool, assessed via population‐level means. For a given trait and a given metric, dots represent the SES values for each pond, crossed circles represent the SES value averaged across ponds, and boxes give the average confidence interval (0.025–0.975) across 1,000 randomizations for each pond. For a given metric, the mean of SES (crossed circle) is significantly different from the null distribution if not embedded within the box, and dots if they have a colored background.

Figure 3 gives the departure of observed *T*‐statistics values from randomized values for each trait. The results were mainly consistent among traits. The mean values of *T*_IP.IC _were lower than expected by chance for all traits, except one, the tail compression index (TCI), which is the trait with the largest ITV (Table 2). Thus, for most ponds (except the ones within or to the right to the boxes), species have nonoverlapping trait distributions, that is, two individuals belonging to a particular population display more similar trait values than two individuals drawn randomly from the pond.

Contrastingly, the values of *T*_IC.IR _did not differ from random expectations on average and for most of the ponds (Figure 3). Communities have trait distributions that are overlapping no more and no less than random ones when considering individual trait values. Two individuals drawn randomly from a given pond are not necessarily more similar or more different than two individuals drawn randomly from the regional pool. Similarly, for all the traits (and for most of the ponds), mean values of *T*_PC.PR_ did not differ from random expectations, meaning that communities have partially overlapping trait distributions when considering population‐level trait values (Figure 3).

Values of *T*_IP.IC _tend to be negatively related to species richness for most of the traits except tail compression index, diameter from nares to snout, and relative diameter of the nares (Supporting information Figure S3).

## 4 DISCUSSION

Here, we proposed one of the first studies to investigate the effect of ITV on animal assemblages. While we observed that intraspecific variability (ITV) is generally lower than interspecific variability (about 30% of total trait variation); we also observed that ITV can be particularly high for some traits such as the tail compression index (>60% in both datasets).

Our results also point to strong effects of internal filtering in shaping tadpole communities. Below, we discuss these results and the discrepancies among traits in the light of species coexistence.

### 4.1 Tadpole traits variability within species

We found about a third of trait variation in tadpole communities from the Atlantic Forest to occur within species, with two traits being more variable within than among species (height of the dorsal fin‐HDF and tail compression index‐TCI). Interestingly, results were similar with a second dataset from Amazonia, another tropical forest in Brazil (Supporting information Appendix S1). This variability was not simply driven by ontogeny, because all the stages presented similar variability in the different traits (Supporting information Figure S2). This is in agreement with the literature on tadpoles, which suggests that morphological characters are highly variable within species because anuran larvae are highly adaptive to their environment (Grosjean, 2005). Grosjean (2005) also found tail traits to be more variable than body traits, as we did. Here, the tail compression index (TCI) presented high levels (69%) of intraspecific variability in both datasets (Atlantic forest and Amazonia). Several studies have also shown that wide morphological variations can be found within a single genus (*Melanophryiniscus*, Baldo et al., 2014), and even single species (Zhao et al., 2017). Interestingly, our results are overall in agreement with decompositions of trait variation performed in plants, as most studies have found between 25% and 33% of trait variation to occur within species (Albert et al., 2010; Messier et al., 2010; Siefert et al., 2015). Recent studies on animal communities have also shown that ITV can be pretty wide for certain organisms such as fishes (Zhao et al., 2014), and less wide for others such as insects (dung beetles, Griffiths et al., 2016). If a third of ITV means that ITV is smaller than variation among species, previous studies have shown that such a nonnegligible amount of ITV may already play a strong role in community assembly and diversity (Albert et al., 2012; Griffiths et al., 2016; Jung, Violle, Mondy, Hoffmann, & Muller, 2010).

### 4.2 Internal filtering more than external filtering shapes tadpole communities

For all traits, we found trait variation in tadpoles to be largely due to local differences, and little to differences among populations or species in different localities or ponds. These results are in agreement with previous studies concluding that morphological variations occur over short distances, and are poorly explained by broad‐scale environmental gradients (Grözinger et al., 2014). Tadpoles from a single species can adapt to the availability of microhabitats or to local conditions (e.g., canopy cover, predation pressure, density‐dependence, Strauβ et al., 2010), which means ITV could be locally high for traits related to their position in the water column (body shape) or to the use of different resource types (Eterovick & Fernandes, 2001; Kopp & Eterovick, 2006). However, this contrasts with the findings of Zhao et al. (2017), who found traits to be also widely variable among populations for a given species.

Contrary to our expectations, we found little support for a contribution of external filters (*T*_IC.IR_ and *T*_PC.PR _not significantly different from randomizations) to the structuration of tadpole communities. For most ponds, the height of the ventral (HVF) fin and relative diameter of the eyes (RDE) showed some signs of external filtering at the individual level (*T*_IC_IR _below random expectations). For the other traits, some of the ponds did too. These signs of external filtering were not visible at the population level (*T*_PC_PR_). This means that the population mean trait values do not differ from the regional mean trait values, but that individual trait values are less variable within populations than at the regional scale. A lower variability in the height of the ventral fin (HVF) and relative diameter of the eyes (RDE) in comparison to the regional pool might be explained by water depth, because shallow ponds harbor only benthic tadpoles (Queiroz, Silva, & Rossa‐Feres, 2015) that have smaller ventral fins and smaller eyes than nektonic ones (Altig & Johnston, 1989; Altig & McDiarmid, 1999). This is not clearly supported by our data (no effect of water depth of trait variability). Alternatively, tadpoles tend to develop large tails and smaller bodies when they are under predation pressure (Van Buskirk & McCollum, 2000; Relyea, 2002), which may also lead to an external filtering for those ponds in which predators occur. We do not have any information on the occurrence of predators, such as fishes, to test this potential explanation.

Contrary to our expectations, we also found strong signs of internal filtering in tadpole communities. *T*_IP.IC _were for the most part lower than expected by chance for all traits except the tail compression index (TCI), thus indicating little overlap of trait distributions among populations within communities. Local processes including biotic interactions and the use of microhabitats is thus key to explaining tadpole assemblages (but see, Kopp & Eterovick, 2006; Strauβ et al., 2010). We acknowledge, however, that the results presented here rely on trait‐based and pattern‐based correlative methods from which processes can only cautiously be inferred (Enquist et al., 2015). The degree of trait variability and the detection of trait overlap within communities also largely depend on the trait under investigation. We discuss these discrepancies in the light of the framework proposed by Turcotte and Levine (2016) to relate ITV with species coexistence.

### 4.3 Implications for tadpoles’ coexistence

**Figure 4:**
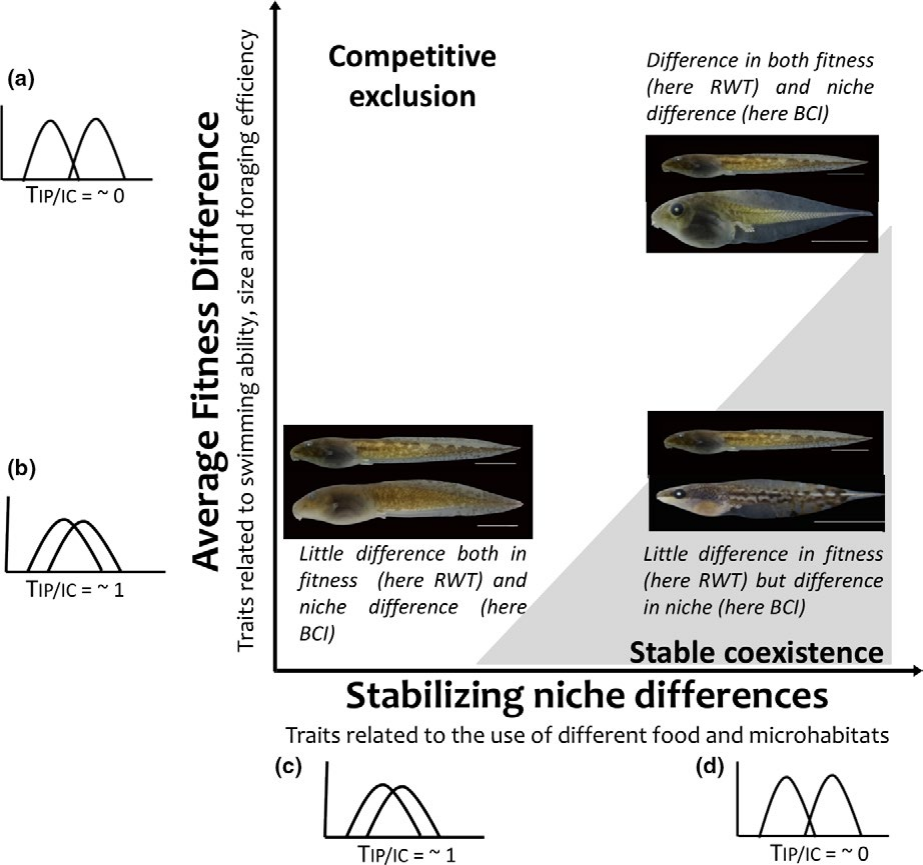
Graphical representation of how coexistence is mediated by intraspecific variability regarding both stabilizing niche differences (*x*‐axis) and average fitness differences (*y*‐axis). After Turcotte and Levine (2016). According to theses authors, stable coexistence (gray area) is predicted when niche differences exceed fitness differences. If not, competitive exclusion (white area) is predicted. (a–d) Give the hypothetical link between trait differences and the corresponding *T*_IP‐IC_ values. Examples show two competing tadpoles with different trait values. Case 1 (bottom left*)*: if both tadpoles have similar swimming capacity due to similar relative width of the tail‐RWT (fitness differences b), and use the same microhabitats due to similar body compression index‐ BCI (niche differences c), one may outcompete the other due to resource limitation. Case 2 (top right*)*: if both tadpoles use different microhabitats due to different body compression index‐BCI (niche differences d), but they have strong differences in swimming capacity due to different relative width of the tail‐RWT (fitness differences a), one may outcompete the other because niche difference does not compensate for fitness advantage of a higher ability to escape. Case 3 (bottom right): if both tadpoles have similar swimming capacity due to similar relative width of the tail‐RWT (fitness differences b), but they use different microhabitats due to different body compression index‐BCI (niche differences d), they can coexist

Turcotte and Levine (2016) proposed that coexistence is enhanced when traits related to stabilizing niche differences are less variable within species than those related to fitness differences (Figure 4).

Coexistence would be promoted when traits related to fitness differences present weak internal filtering (high *T*_IP.IC _values), which means individuals from different species may have similar trait values, and competitive outcome depends on the individuals (e.g., asymmetric competition, Le Bagousse‐Pinguet et al., 2014). We found for instance, high intraspecific variation and reduced internal filtering for tail compression index (TCI), which relates to swimming ability, a key component of fitness differences (escape from predator). This is less clear, however, for other traits that may relate to fitness differences such as the relative width of the tail (RWT) (swimming ability and escape from predator) and distance from nares to snout (DNS) and relative diameter of the nares (RDN) (foraging efficiency and food acquisition), that present stronger internal filtering (Figure 3). Coexistence would also be favored when traits related to niche differences present strong internal filtering (low *T*_IP.IC _values), which means individuals from a given species can specialize in a type of resource/microhabitat, which helps in avoidance of interspecific competition. This is what we found for the body compression index (BCI) and the relative diameter of the eyes (RDE) (strong internal filtering and medium intraspecific trait variation), and is less true for height of the dorsal and ventral fin (HDF and HVF, respectively) that present intermediate levels of filtering; these four traits relate to the use of different food and microhabitats (position in the water column), and could thus be associated with stabilizing niche differences.

Examining the relationship between *T*_IP.IC _and species richness mainly confirms these trends (Supporting information Figure S3). In species‐rich ponds, overlaps in trait distributions are slighter, which corresponds to increased “niche packing” (increased internal filtering, i.e., lower *T*_IP.IC _values), for traits related to stabilizing niche differences (relative diameter of the eyes ‐ RDE, body compression index—BCI, height of the dorsal fin‐HDF and height of the ventral fin‐HVF), and higher for traits related to fitness differences (tail compression index—TCI). For the distance from nares to snout (DNS) and the relative diameter of nares (RDN), there is no relationship between *T*_IP.IC_ and species richness, and for relative width of the tail (RWT) the relationship is negative (smaller overlaps in richer ponds).

Discrepancies among traits suggest that more attention should be paid to the different traits and their potential contribution to different assembly processes (Spasojevic & Suding, 2012). Though the ecological function attributed to each trait represents the best of our knowledge regarding the biology of neotropical tadpoles (Queiroz et al., 2015; Rossa‐Feres et al., 2015; Sousa et al., 2015, 2014), attributing one trait to one axis or the other is not straightforward (Kraft et al., 2015). For instance, traits reflecting foraging efficiency via detection of chemicals or swimming ability may contribute to both predator escape and the use of various microhabitats, therefore contributing to both fitness differences and niche differences. This may also explain the conflicting results obtained in recent studies. While some studies support equalizing mechanisms as drivers of community assembly (more trait overlap in species‐rich communities, for example, Le Bagousse‐Pinguet et al., 2014; Li et al., 2018), others better support niche theory (less trait overlap in species‐rich communities, for example, Siefert et al., 2015; Kumordzi et al., 2015) or neutral theory (no change, Bastias et al., 2017). These studies are, however, all focused on plant communities, communities that are typically richer than the ones presented here (3‐12 species per pond; e.g., Bastias et al., 2017).

### 4.4 Sampling issues regarding ITV in tadpoles

Sampling design can strongly influence a study's results. Plot size, number of plots, number of measured individuals may strongly influence trait variation and its decomposition into intraspecific and interspecific (and intraspecific and interpopulation) components (Albert, 2015; Albert, Grassein, Schurr, Viellendent, & Violle, 2011; Siefert et al., 2015). The selection of species and individuals to be measured may also strongly influence our ability to detect assembly processes (Bentley et al. submitted). Violle, Borgy, and Choler (2015) advocated relaxing standardized protocols when applying a trait‐based approach to plant community ecology. Rather than aiming to minimize intraspecific variability, the goal is to accurately quantify intraspecific variation within communities, which requires a random selection of individuals (de Bello et al., 2011).

Tadpoles are not plants, and protocols have to be adjusted to their specificities if we are to disentangle the role of ITV in assembly processes in natural tadpole assemblages. Firstly, due to the typical anuran life history (large egg clutches and high juvenile mortality rates, Duellman & Trueb, 1994), species abundances are very disparate, with up to several thousands of coexisting individuals—potentially siblings—in a single pond (Hoff et al., 1999). Accurate estimations of ITV thus probably require a minimum number of individuals to be measured. To make accurate estimates of species mean trait values, Hulshof and Swenson (2010) recommend measuring traits on 10–20 individuals per community for tropical trees. Griffiths et al. (2016) encourage the measurement of 30–60 individuals for dung beetles. Here, we picked 20 individuals mainly for logistical reasons. Secondly, by definition tadpoles are larvae and their morphology is constantly changing (Grosjean, 2005). Here, we reduced the ontogenetic variation by focusing on some developmental stages (27–37), considered to be a developmental “climax” period, when changes in body parts of the tadpoles are isometric and when they are best suited for morphological intraspecific and interspecific comparisons, with less impact of ontogeny (Gosner, 1960; Grosjean, 2005; Wassersug, 1973). In our case, traits were as variable within as among stages (see Figure S2). This means, however, that we only studied a portion of the ponds' filter‐feeding and grazing assemblages; the stages we did not measure, along with other taxa, may also influence trait variability, and we may have underestimated the degree of overlap between species (Violle et al., 2012). Another option could have been to control for developmental stages in the statistical analyses, but this would require balancing the sampling design among stages (Zhao et al., 2017).

Overall, our results emphasize the importance of incorporating both intraspecific and interspecific trait differences, and of focusing on traits related to both stabilizing niche and fitness differences in order to better understand how trait variation relates to species coexistence. Detecting trait overlap and niche packing may largely depend on the trait under investigation, and on whether or not ITV is accounted for. Future studies are needed to explore the different sources of variability in tadpole traits, and how they relate to community assembly. If *T*‐statistics offer a new avenue to account for ITV, while investigating patterns associated with assembly processes, there is also a need for studies of animals and plants to move beyond pattern‐based and correlative assessments (Enquist et al., 2015).

## CONFLICT OF INTEREST

None declared.

## AUTHOR CONTRIBUTIONS

MXJ compiled the data. MXJ and CHA analyzed the data. MXJ, CHA, NM and DRF interpreted results. MXJ and CHA wrote the paper with contribution from all co‐authors.

## Data Availability

The data used in analyses for this manuscript will be available. Tadpoles ocurrence and morphological data: Dryad https://doi.org/10.5061/dryad.n410g85.
