# Supplementary material for: Intraspecific and interspecific trait variability in tadpole meta‐communities from the Brazilian Atlantic rainforest
Source: Ecol Evol. 2019 Mar 13;9(7):4025–37. doi: 10.1002/ece3.5031 (PMC6467852; doi:10.1002/ece3.5031)
Supplement: Supplementary file 1 [file ECE3-9-4025-s001.docx]

**Supplementary Material**

**Table S1:** Environmental variables for the 43 ponds from Atlantic Forest. Communities with more than one species and more than one individual per species for each population are selected to perform T-statistic (**♦**). Classification of amount of aquatic vegetation 1 = low; 2 = medium; 3 = high. The sequence of substrate type indicates their predominance in descending order. Abbreviations: BER = Bertioga; ITA = Itanhaém; JUR = Jureia/Iguape; UPI = Ubatuba/Picinguaba; Lat = latitude; Long = longitude; SR = species richness; AB = abundance; NSM = number of species measured; TNIM = total number of individuals measured; MNIMS = minimal number of individuals measured by species; WAD = water depth; LG = length; WD = width; AAV = amount of aquatic vegetation; CC = canopy cover.

| **Site** | **Lat** | **Long** | **SR** | **AB** | **NSM** | **TNSM** | **MNIMS** | **WAD**  **(m)** | **LG**  **(m)** | **WD**  **(m)** | **AAV** | **CC**  **(%)** | **Substrate type** |
| --- | --- | --- | --- | --- | --- | --- | --- | --- | --- | --- | --- | --- | --- |
| **BER2 ♦** | -23.7433 | -45.8655 | 4 | 396 | 4 | 23 | 2 | 1.5 | 172.0 | 52.1 | 3 | 0 | sand = mud > boulders |
| **BER3 ♦** | -23.740 | -45.7673 | 4 | 175 | 3 | 11 | 2 | 1.1 | 110.2 | 6.2 | 3 | 0 | sand = mud > leaf litter |
| **BER4** | -23.7438 | -45.7634 | 2 | 2415 | 1 | 7 | 7 | 1.1 | 20.0 | 8.0 | 3 | 54 | sand = mud > leaf litter |
| **BER6** | -23.7355 | -45.9312 | 11 | 199 | 5 | 16 | 1 | 1.2 | 30.0 | 8.0 | 3 | 69 | sand > mud > boulders = leaf litter |
| **BER7** | -23.7544 | -45.9265 | 4 | 312 | 1 | 7 | 7 | 1.1 | 16.0 | 16.0 | 3 | 64 | mud > sand > leaf litter |
| **BER9** | -23.762 | -45.9163 | 7 | 95 | 3 | 11 | 1 | 1.6 | 100.0 | 3.5 | 3 | 33 | mud > sand > leaf litter |
| **BER10** | -23.7265 | -45.7528 | 10 | 510 | 4 | 16 | 1 | 0.4 | 50.0 | 20.0 | 3 | 0 | sand = mud > leaf litter |
| **BER11 ♦** | -23.7336 | -45.7476 | 5 | 443 | 2 | 18 | 8 | 1.1 | 50.0 | 10.0 | 3 | 78 | sand = mud > leaf litter |
| **BER12** | -23.7362 | -45.7471 | 5 | 622 | 2 | 7 | 1 | 0.6 | 50.0 | 30.0 | 3 | 0 | sand = mud |
| **ITA1** | -24.1986 | -46.9394 | 12 | 770 | 6 | 32 | 1 | 0.3 | 69.0 | 2.5 | 3 | 0 | sand = mud = leaf litter > cobbles = gravel |
| **ITA2** | -24.2173 | -46.8946 | 2 | 371 | 1 | 8 | 8 | 0.4 | 65.0 | 90.0 | 3 | 0 | leaf litter > sand = mud |
| **ITA3** | -24.2249 | -46.8944 | 1 | 137 | 1 | 8 | 8 | 1.5 | 118.0 | 35.0 | 3 | 0 | sand = mud = leaf litter > cobbles = gravel |
| **ITA4** | -24.2341 | -46.9264 | 2 | 4 | 1 | 2 | 2 | 0.5 | 30.0 | 2.5 | 3 | 81 | mud = leaf litter |
| **ITA5** | -24.2407 | -46.9144 | 6 | 122 | 4 | 15 | 1 | 0.3 | 49.0 | 7.8 | 3 | 0 | mud > sand > boulders |

**Table S1:** Continued.

| **Site** | **Lat** | **Long** | **SR** | **AB** | **NSM** | **TNSM** | **MNIMS** | **WAD**  **(m)** | **LG**  **(m)** | **WD**  **(m)** | **AAG** | **CC**  **(%)** | **Substrate type** |
| --- | --- | --- | --- | --- | --- | --- | --- | --- | --- | --- | --- | --- | --- |
| **ITA6** | -24.2412 | -46.9313 | 1 | 313 | 1 | 2 | 2 | 0.6 | 14.8 | 4.6 | 3 | 51 | sand > mud = leaf litter |
| **ITA7** | -24.2463 | -46.9311 | 3 | 357 | 2 | 8 | 1 | 2.0 | 195.0 | 16.0 | 3 | 0 | sand = mud |
| **ITA8** | -24.2326 | -46.9265 | 1 | 246 | 1 | 7 | 7 | 1.6 | 14.3 | 8.2 | 3 | 70 | mud > leaf litter |
| **JUR1** | -24.4444 | -47.0903 | 11 | 1141 | 5 | 16 | 1 | 1.0 | 50.0 | 41.3 | 3 | 65 | leaf litter > sand = mud |
| **JUR2** | -24.4621 | -47.1104 | 11 | 772 | 6 | 27 | 1 | 0.8 | 50.0 | 9.3 | 3 | 74 | mud = leaf litter > sand |
| **JUR3** | -24.4747 | -47.1266 | 2 | 30 | 1 | 6 | 6 | 4.0 | 50.0 | 14.2 | 3 | 0 | sand = mud |
| **JUR4 ♦** | -24.5338 | -47.2039 | 6 | 1108 | 4 | 22 | 2 | 1.0 | 95.0 | 12.0 | 3 | 0 | sand = mud > leaf litter |
| **JUR6** | -24.5728 | -47.2478 | 4 | 513 | 1 | 8 | 8 | 0.7 | 51.2 | 22.1 | 3 | 43 | mud > sand > boulders = leaf litter |
| **UPI1 ♦** | -23.3701 | -44.8170 | 6 | 94 | 3 | 16 | 3 | 0.3 | 13.77 | 6.4 | 1 | 78.4 | mud > leaf litter > boulders = clay |
| **UPI2** | -23.3591 | -44.8336 | 12 | 2084 | 9 | 51 | 1 | 0.5 | 115.3 | 48.0 | 2 | 8.4 | mud > leaf litter |
| **UPI3** | -23.3607 | -44.8335 | 3 | 23 | 1 | 4 | 4 | 0.7 | 12 | 23.4 | 2 | 64.9 | mud > leaf litter > sand = boulders |
| **UPI4 ♦** | -23.3556 | -44.8158 | 3 | 15 | 2 | 12 | 2 | 0.2 | 1.56 | 1.0 | 1 | 0 | clay = mud = leaf litter |
| **UPI5** | -23.3459 | -44.8483 | 8 | 2243 | 8 | 59 | 1 | 0.3 | 11.69 | 5.3 | 1 | 74.15 | mud = leaf litter > sand |
| **UPI6 ♦** | -23.3571 | -44.8508 | 5 | 144 | 2 | 15 | 5 | 0.3 | 3.62 | 0.8 | 1 | 74.15 | mud > sand = leaf litter > clay |
| **UPI7** | -23.3640 | -44.8265 | 1 | 15 | 1 | 10 | 10 | 0.6 | 7.8 | 4.2 | 2 | 95.8 | mud > leaf litter |

**Table S1:** Continued.

| **Site** | **Lat** | **Long** | **SR** | **AB** | **NSM** | **TNSM** | **MNIMS** | **WAD**  **(m)** | **LG**  **(m)** | **WD**  **(m)** | **AAG** | **CC**  **(%)** | **Substrate type** |
| --- | --- | --- | --- | --- | --- | --- | --- | --- | --- | --- | --- | --- | --- |
| **UPI8** | -23.3633 | -44.8214 | 1 | 207 | 1 | 19 | 19 | 0.6 | 17.7 | 3.2 | 2 | 95.3 | mud > leaf litter > gravel = sand |
| **UPI9** | -23.3583 | -44.8172 | 1 | 42 | 1 | 18 | 2 | 0.2 | 10.9 | 6.9 | 2 | 86.1 | mud > leaf litter |
| **UPI10** | -23.3769 | -44.8185 | 1 | 32 | 1 | 18 | 18 | 0.9 | 13.8 | 3.8 | 1 | 91.9 | mud > leaf litter > sand |
| **UPI11** | -23.3628 | -44.8249 | 1 | 1 | 1 | 1 | 1 | 0.2 | 13.6 | 2.4 | 1 | 97.5 | mud = leaf litter > sand |
| **UPI12** | -23.3538 | -44.8149 | 3 | 82 | 1 | 8 | 8 | 0.8 | 7.06 | 3.0 | 1 | 96.1 | mud > clay = leaf litter |
| **UPI13 ♦** | -23.3588 | -44.8335 | 12 | 6322 | 9 | 65 | 5 | 0.4 | 17.2 | 7.1 | 2 | 0 | mud = leaf litter > sand |
| **UPI14** | -23.3522 | -44.8533 | 3 | 176 | 1 | 3 | 3 | 0.5 | 50.0 | 50.0 | 3 | 40 | mud > leaf litter |
| **UPI15 ♦** | -23.3602 | -44.8498 | 5 | 2994 | 4 | 32 | 7 | 1.3 | 180.0 | 6.5 | 3 | 58 | mud > leaf litter > boulders = gravel = sand |
| **UPI16** | -23.3589 | -44.8507 | 8 | 262 | 4 | 10 | 1 | 0.6 | 18.0 | 5.1 | 3 | 69 | sand = mud = leaf litter > boulders = gravel |
| **UPI17** | -23.3593 | -44.8327 | 2 | 2290 | 1 | 3 | 3 | 0.1 | 6.9 | 8.5 | 3 | 0 | mud > sand |
| **UPI18** | -23.3563 | -44.8266 | 4 | 8 | 1 | 5 | 5 | 0.6 | 100.0 | 2.7 | 3 | 72 | leaf litter > sand |
| **UPI19 ♦** | -23.3447 | -44.8542 | 4 | 312 | 2 | 9 | 4 | 2.0 | 180.0 | 17.2 | 3 | 37 | mud > sand |
| **UPI20** | -23.3644 | -44.8324 | 5 | 303 | 4 | 26 | 1 | 0.5 | 80.0 | 50.0 | 3 | 0 | sand = mud |
| **UPI21 ♦** | -23.3564 | -44.8536 | 4 | 250 | 4 | 17 | 2 | 0.3 | 40.0 | 20.0 | 3 | 82 | leaf litter > sand |

**Table S2:** Larval anuran species found in 43 ponds in the Atlantic Rain Forest lowlands (São Paulo state, Brazil). Number of individuals net-caught (all development stages, SISBIOTA project) and measured (development stages 27- 37) individuals. Taxonomy and nomenclature follow Frost (2018).

| **Family** | **Species** | **N of net-caught individuals** | **N of measured individuals** |
| --- | --- | --- | --- |
| Bufonidae | *Rhynella ornata* | 3039 | 36 |
| Hylidae | *Aplastodiscus eugenioi* | 43 | 63 |
|  | *Dendropsophus berthalutzae* | 33 | 18 |
|  | *Dendropsophus elegans* | 230 | 42 |
|  | *Dendropsophus gisleris** | 3 | 3 |
|  | *Dendropsophus microps* | 18 | 6 |
|  | *Dendropsophus minutus* | 165 | 10 |
|  | *Dendropsophus werneri* | 72 | 14 |
|  | *Boana albomarginata* | 3477 | 97 |
|  | *Boana faber* | 1505 | 15 |
|  | *Boana semilineata* | 3176 | 29 |
|  | *Itapothyla langsdorffii* | 3159 | 16 |
|  | *Scinax argyreornatus* | 349 | 23 |
|  | *Scinax hayii* | 452 | 27 |
|  | *Scinax littoralis** | 125 | 1 |
|  | *Scinax perereca* | 124 | 15 |
|  | *Scinax perpusillus** | 1 | 0 |
|  | *Scinax trapicheiroi* | 450 | 49 |
|  | *Scinax tymbamirim* | 1078 | 119 |
|  | *Trachycephalus mesophaeus* | 444 | 19 |
| Leptodactylidae | *Leptodactylus latrans* | 9272 | 20 |
|  | *Physalaemus atlanticus* | 45 | 46 |
|  | *Physalaemus cuvieri** | 1 | 0 |
| Microhylidae | *Chiasmocleis carvalhoi** | 9 | 2 |
|  | *Elachistocleis bicolor* | 26 | 13 |

***** *not included in the statistical analysis*


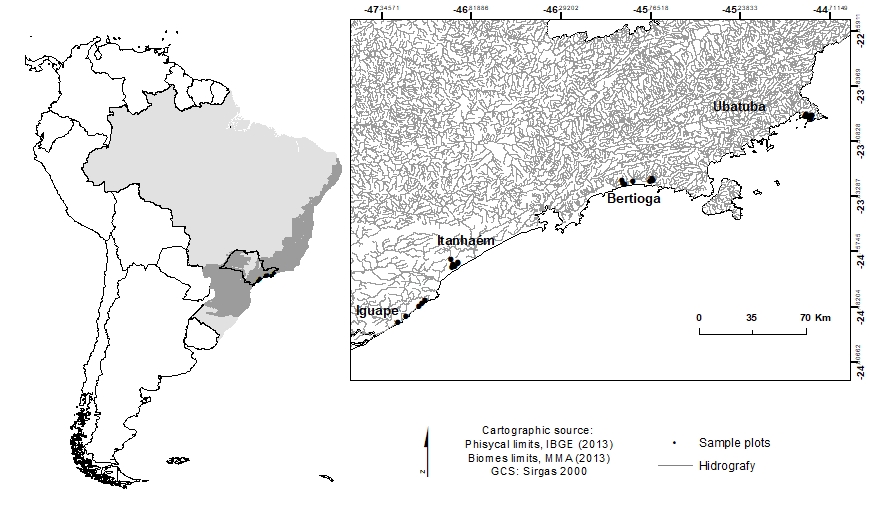


**Figure S1:** Geographical distribution of the 43 ponds in Atlantic Forest, sampled in four localities (Bertioga, Iguape, Itanhaém, and Ubatuba), in São Paulo state, Brazil


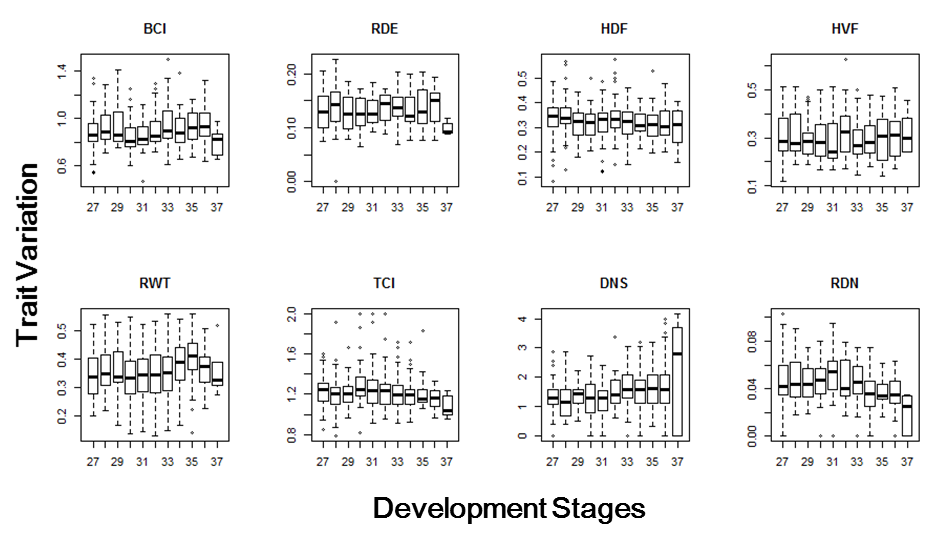


**Fig S2:** Variance of tadpoles traits (BCI = Body compression index; RDE = Relative diameter of the eyes; HDF= Relative height of the dorsal fin; HVF = Relative height of the ventral fin; RWT = Relative width of the tail; TCI = Tail compression index; DNS = Distance from nares to snout; RDN = Relative diameter of the nares) according to development stages, considering all species together.


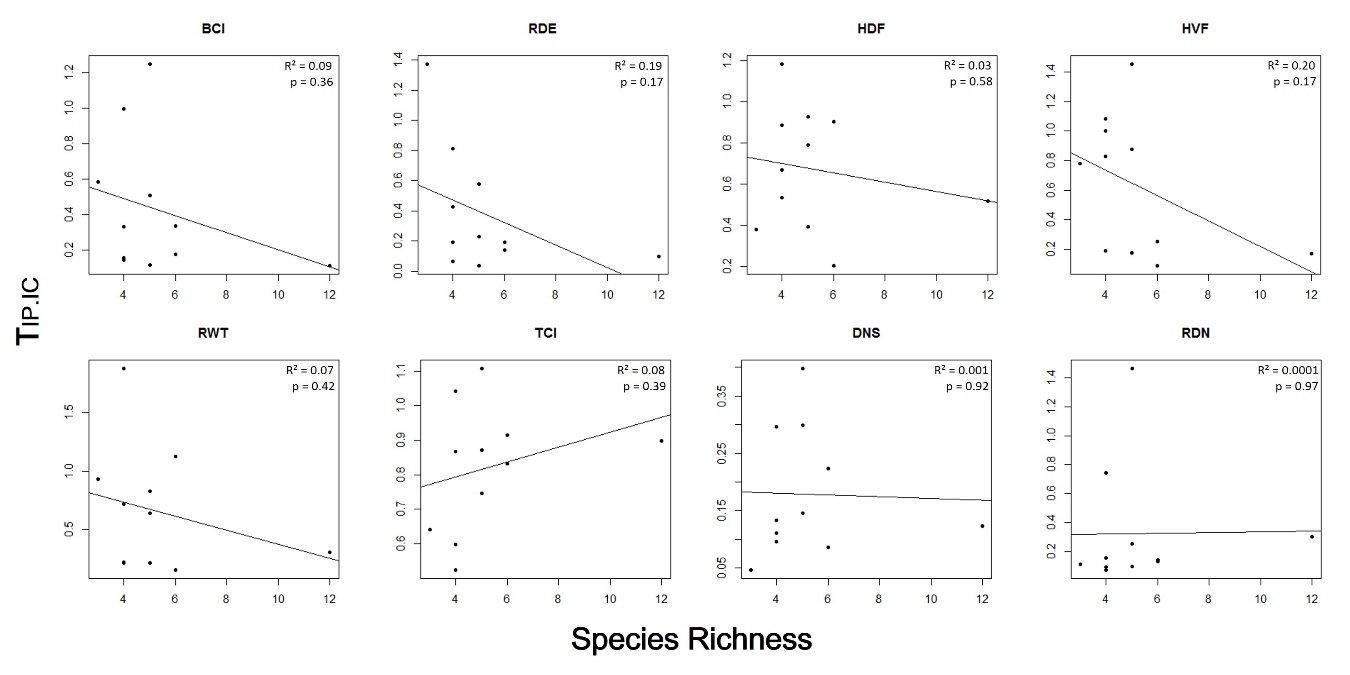


**Figure S3:** Relationship between T_IP.IC_ and species richness in 11 ponds, for each tadpole trait (BCI = Body compression index; RDE = Relative diameter of the eyes; HDF= Relative height of the dorsal fin; HVF = Relative height of the ventral fin; RWT = Relative width of the tail; TCI = Tail compression index; DNS = Distance from nares to snout; RDN = Relative diameter of the nares).

**Supplementary – Amazonia dataset**

We sampled tadpoles communities in three different localities (Iranduba, Manaus, and Presidente Figueredo cities) in Amazonas state, Northern Brazil. Tadpoles from the Amazon Forest were deposited in “Paulo Bührnheim” collection from the Universidade Federal do Amazonas (CZPB collection - UFAM), Manaus, Amazonas, Brazil. From that collection, we randomly selected and measured, from 1 to 10 individuals (depending on availability) in the developmental stages 33-37 (Gosner 1960), for each species occurring in each pond. In the total, we measured traits of 60 tadpoles from 13 species (Table S3), found in 31 ponds.

For single-trait analysis the tail compression index (TCI) showed a high contribution of intraspecific variability (>85%) while for all remaining traits the contribution of intraspecific variability to total trait variation was low (<25%, Table S4). For multi-trait analysis, the trait variability within-species was mainly structured by a first axis (56% of variation) driven by tail compressed index (TCI), and by a second axis (13% of variation) driven by the height of dorsal fin (Fig. S4a). Between species, the trait variability was structured by a first axis (62% of variance) driven by relative width of the tail (RWT), distance from nares to snout (DNS), height of ventral fin (HVF), relative diameter of the nares (RDN), and diameter of eyes (RDE), and a second axis (17% of variation) driven by body compression index (BCI) and height of dorsal fin (HDF; Fig. S4b).

**Table S3:** Larval anuran species found in 31 ponds in Central Amazon (Amazonas state, Brazil). Numbers of net-caught in project SISBIOTA (all stages of development) and measured (33 to 37 development stages) individuals. Taxonomy and nomenclature follow Frost (2018).

| **Family** | **Species** | **N of net-caught individuals** | **N of measured individuals** |
| --- | --- | --- | --- |
| Aromobatidae | *Allobates femoralis** | 1 | 0 |
|  | *Allobates sumtuosus* | 533 | 30 |
| Bufonidae | *Amazophrynella manaos* | 11 | 3 |
|  | *Rhinella proboscidea** | 22 | 1 |
| Dendrobatidae | *Ameerega hahneli** | 2 | 0 |
| Hylidae | *Boana cinerascens** | 1 | 1 |
|  | *Boana fasciata* | 37 | 4 |
|  | *Boana raniceps** | 3 | 1 |
|  | *Dendropsophus brevifrons* | 4 | 2 |
|  | *Dendropsophus walfordi* | 27 | 9 |
|  | *Osteocephalus taurinus* | 7 | 2 |
|  | *Phyllomedusa bicolor** | 26 | 1 |
|  | *Sphaenorhynchus carneus** | 1 | 1 |
| Leptodactylidae | *Leptodactylus rhodomystax* | 24 | 3 |
| Microhylidae | *Chiasmocleis hudsoni* | 2 | 2 |

***** *not included in the statistical analysis*

**Table S4:** Decomposition of tadpole trait from Amazon in intra- and interspecific components. Relative proportions of variance are given for each of eight tadpole traits separately (after a re-sampling procedure), an average across traits (single-trait analysis), and for all traits together (multi-traits analysis). Square brackets represent the 95% confidence intervals from the resampling procedure. The largest (intra- or interspecific) component is in bold.

| **Functional traits** | **Amazon** | |
| --- | --- | --- |
|  | **Intraspecific**  **variability** | **Interspecific**  **variability** |
| Body compression index | 0.250 [0.246 - 0.253] | **0.750 [0.747 - 0.754]** |
| Relative diameter of the eyes | 0.095 [0.088 - 0.101] | **0.905 [0.899 - 0.911]** |
| Relative height of the dorsal fin | 0.165 [0.163 - 0.166] | **0.835 [0.833 - 0.837]** |
| Relative height of the ventral fin | 0.231 [0.230 - 0.233] | **0.768 [0.767 - 0.769]** |
| Relative width of the tail | 0.017 [0.017 - 0.018] | **0.982 [0.982 - 0.984]** |
| Tail compression index | **0.868 [0.859 - 0.877]** | 0.131 [0.122 - 0.140] |
| Distance from nares to snout | 0.077 [0.072 - 0.082] | **0.923 [0.917 - 0.928]** |
| Relative diameter of the nares | 0.136 [0.133 - 0.139] | **0.864 [0.860 - 0.867]** |
| **Average of single-trait analysis** | 0.229 [0.226 - 0.234] | 0.769 [0.766 - 0.773] |
| **Average of multi-trait analysis** | 0.154 [0.153 - 0.155] | 0.847 [0.845 - 0.848] |


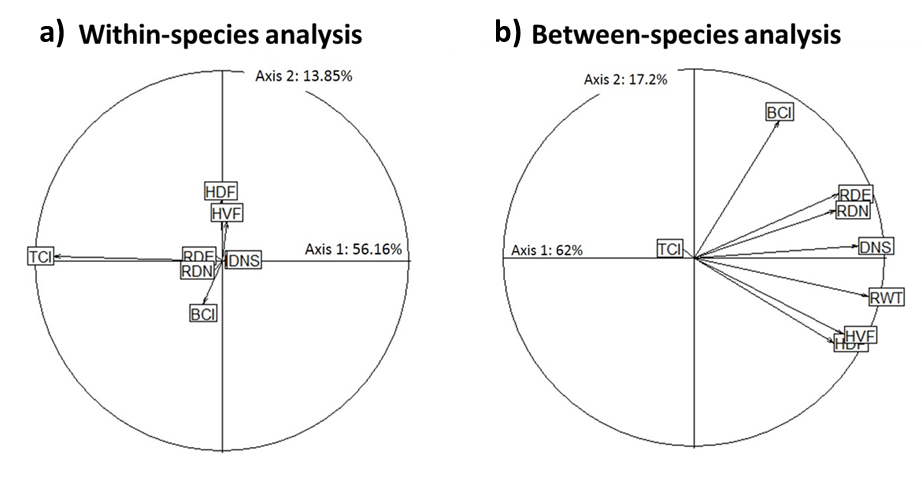


**Figure S4:** Correlation circles regarding the first two axes of (a) within-species and (b) between-species PCA on functional traits for tadpoles communities from Amazon. Abbreviations: BCI = Body compression index; RDE = Relative diameter of the eyes; HDF= Relative height of the dorsal fin; HVF = Relative height of the ventral fin; RWT = Relative width of the tail; TCI = Tail compression index; DNS = Distance from nares to snout; RDN = Relative diameter of the nares.

**Literature cited**

Frost, D. R. (2018) Amphibian Species of the World: an online reference. Version 6.0 (Date of access). Electronic Database accessible at http://research.amnh.org/herpetology/amphibia/index.html. American Museum of Natural History, New York, USA.
